# Dietary yolk supplementation decreases rates of yolk deposition in Japanese quail

**DOI:** 10.1002/jez.2653

**Published:** 2022-09-06

**Authors:** Kristen J. Navara, Kylie Graden, Mary T. Mendonça

**Affiliations:** ^1^ Department of Poultry Science The University of Georgia Athens Georgia USA; ^2^ Department of Biological Sciences Auburn University Auburn Alabama USA

**Keywords:** bird, rapid yolk deposition, yolk precursors, yolk size

## Abstract

Generation of egg yolk by birds requires the synthesis and deposition of large amounts of protein and lipid, and is often accompanied by the incorporation of additional physiological mediators. While there has been much work examining the relative quantities of yolk components, as well as potential adaptive patterns of their allocation, we still do not have a full understanding of what controls yolk formation and composition. Once ovarian follicles are recruited into the preovulatory hierarchy, the yolk is deposited in concentric rings, with one ring deposited per day. Previous studies have shown that there is substantial interspecific and intraspecific variation in the number of rings in yolks, and thus the number of days it took those yolks to grow. We hypothesized that the ability to grow follicles to maturity quickly is limited by the availability of materials to make yolk precursors in the female, either in body reserves or in dietary access. To test this, we supplemented the diets of Japanese quail with hard‐boiled chicken yolk and examined the influences of treatment and female body condition on follicle growth rates. Contrary to predictions, females with higher body condition indices produced yolks that grew more slowly, and yolks from supplemented birds grew more slowly than controls. These results indicate that females can modulate the rate of yolk incorporation into developing follicles, and that an energy balance that is too high may not be optimal for the fast growth of developing ovarian follicles.

## Research Highlights

- Dietary supplementation with egg yolk decreases the rate of yolk deposition in quail.
- Quail with high body condition indices deposit yolk more slowly.

## Graphical abstract

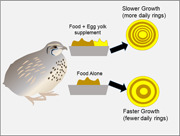

## 1 INTRODUCTION

For birds, a significant portion of reproductive investment lies in the formation of eggs. Yet this investment is necessary because embryos are separated from parents by the eggshell for the duration of embryonic development, and the contents of the egg are the main determinant of the survival of the offspring. During yolk deposition, many physiologically active components that can influence offspring quality and survival are also deposited into eggs, including energy reserves such as lipids and proteins (Johnson, 2015), and other physiologically relevant substances, such as antioxidants (Blount et al., 2000; Navara et al., 2006), hormones (Gil, 2008), and immunoglobulins (Grindstaff et al., 2003). We now know that females vary the quantities of each of these components according to environmental and social conditions (Jenni‐Eiermann et al., 2020; Muriel et al., 2019), and that such variation substantially influences the resulting behavior and physiology of offspring (Groothuis & von Engelhardt, 2005; Williams & Groothuis, 2015). Yet, we still lack a complete understanding of how these egg components, particularly those in the yolk, are deposited. Further, despite over a century of basic research on egg formation, the factors that influence the most basic characteristics of the yolk itself, including its rate of growth and its final size, remain unclear.

The bulk of egg formation occurs when small yellow ovarian follicles are recruited into the preovulatory hierarchy. These follicles then enter into a stage of “rapid yolk deposition” (RYD) when concentric rings of yolk are deposited in an accelerated fashion. Domestic hens can deposit up to 2 g of yolk into a hierarchical follicle per day, increasing the volume from 0.2 to 35 g over a 7−10‐day period (Johnson, 2015; Walzem et al., 1994). The yolk contains 65% lipids and 27% phospholipids by weight, and the major yolk precursors, vitellogenin and very low‐density lipoprotein (VLDL), originate in the liver, which transitions during this time from producing generic VLDL to VLDLy. VLDLy is triacylglycerol‐rich and reduced in size for passage through the granulosa basal lamina surrounding the oocyte (Walzem et al., 1994). These precursors pass through circulation to the ovary, where they are internalized in follicles through receptor‐mediated endocytosis.

Yolk is deposited in rings that alternate in color between white and yellow. When yellow yolk, which is deposited during the day, is viewed through a microscope, it consists of large spheres that are high in fat and carotenoids, while white yolk, which is deposited at night, contains smaller spheres that contain few carotenoids (Riddle, 1911; Romanoff & Romanoff, 1949). One light ring and one dark ring together represent a single day of growth, and staining these rings, either by feeding birds with a lipid‐soluble dye such as Sudan Black, or staining collected eggs with potassium dichromate, allows us to count the number of days the yolk spent in the RYD phase. The number of days spent in RYD varies dramatically among species. Songbird yolks complete their growth in as little as 3−4 days (Young & Badyaev, 2004), chicken and quail yolks take a bit longer (4−6 days for quail and 7−10 days for chicken for full follicular growth) (Grau, 1976; Johnson, 2015), and seabirds take substantially more time to form yolks, an example being glaucous gulls, which take 12 days of RYD to finish yolk development (Roudybush et al., 1979).

While the average time of yolk formation has now been established for many species, there is also within‐species variation in this timing that has not yet been explained. For example, the observed variation of 2 days in quail yolks (Grau, 1976) means that some yolks are developing nearly 40% faster than others. Further, not only is there substantial variation between the eggs produced by different females, but in seabirds it appears that this timing can vary within the same female and even within eggs of a single clutch (Roudybush et al., 1979), though this was never, to our knowledge, examined statistically. What might account for this variation? Do differences in the number, and thus also thickness, of rings represent total differences in energy contribution or spreading of the same contribution over a longer period of time? Does the rate of yolk deposition influence the deposition of other physiological mediators, such as androgens and immunoglobulins? And finally, what are the consequences of these different rates of follicle growth on developing offspring? Before we can answer most of these questions, we first need to understand what accounts for both inter‐ and intraspecific variation in the number of yolk rings deposited by females.

In our study, we aimed to address the first question by testing factors that influence the rate of ovarian follicle growth in Japanese quail. Several existing studies indicate that yolk precursors may be limited resources that are expensive to produce and/or deposit. Work in European starlings showed that females with preovulatory hierarchies that were larger had lower levels of vitellogenin and VLDL in their blood, suggesting that these precursors are depleted as follicles get larger (Challenger et al., 2001). In great tits, food supplementation increased yolk mass (Ruuskanen et al., 2016); in tree swallows, insect prey availability 1−3 days before egg‐laying (in the middle of RYD) predicted yolk mass (Ardia et al., 2006), and a large body of work in poultry indicates that dietary intake of proteins and lipids are key determinants of yolk size (Gardner & Young, 1972; Hartmann & Wilhelmson, 2001; Whitehead et al., 1993). Yet while these studies show the effects of diet on total yolk deposited, it remains unclear whether the *rate* of yolk deposition, and thus the rate of follicle growth, is dependent on dietary intake or diet composition.

Broiler breeder chickens that were fed ad libitum had a higher incidence of simultaneous development of ovarian follicles, which could indicate that they had a higher rate of yolk deposition compared to hens that were feed restricted (Yu et al., 1992). Adding fat to the diets of chickens in the form of linseed oil increases egg mass (Grobas et al., 2001), and Bacon and Cherms (1968) indicated that the weight of the egg may relate to the rate of follicle development. However, another study showed that feed restriction in broiler breeder chickens had the opposite effect; follicles grew faster when feed was restricted (Watson, 1975), indicating some intraspecific variability in how feed restriction influences the process of yolk deposition. Further, in three other studies of chickens and turkeys, feed restriction did not influence the rate of follicle growth (Bruggeman et al., 2005; Hocking et al., 1987; Robinson et al., 1993). In addition, the relationships between body weight and the rate of follicle growth are virtually unstudied. There is evidence that body size relates to the rate of follicle growth; Japanese quail selected for larger body sizes had slower rates of follicle growth than the control line (Bacon et al., 1973), but in turkeys, there was a positive linear relationship between body weight and the number of yellow follicles, indicating more active yolk deposition (Hocking, 1992). Therefore, it is still unclear whether the dietary quantity and quality or body weight influence the rate of ovarian follicle growth.

We hypothesized that available nutritional resources, either available via energetic reserves (measured via female body condition) or acquired through dietary intake, would influence not only yolk size, but also the rate of ovarian follicle growth. We tested this hypothesis in quail by providing daily supplements of hard‐boiled chicken egg yolks to breeding pairs of quail and comparing the numbers of growth rings and the thicknesses of the rings in yolks produced by those females with those produced by a set of control quail. In addition, in control quail, we tested whether maternal body condition predicted the number of growth rings and the thicknesses of the rings in egg yolks. We predicted that, if our first hypothesis is correct, female quails in better body condition would produce yolks with fewer growth rings (indicating faster growth), and that birds supplemented with chicken egg yolk would produce yolks that grew faster than controls.

## 2 MATERIALS AND METHODS

### 2.1 Husbandry and experimental design

For this experiment, we were kindly allowed access to the breeding stock of Japanese quail, *Coturnix japonica*, housed at the Poultry Research Facility at the University of Georgia. This facility has been approved by the UGA Institutional Animal Care and Use Committee for the housing of poultry, including quails. The birds were pair‐housed in breeding cages with a light cycle of 16 h light:8 h dark and, through the duration of the experiment, they were provided ad libitum access to water and a quail diet formulated by nutritionists at the University of Georgia (Table 1). All birds were the same age. At the start of the experiment, we measured the body weights of 60 female quails using a digital scale (VWR‐500P balance, accuracy = 0.01 g) and measured tarsus lengths using digital calipers (accuracy = 0.02 mm). Pairs were then randomly assigned to one of two treatment groups using a random number generator: the first group received dietary supplementation with approximately 18 g of hard‐boiled chicken egg yolk once daily. The second group remained unsupplemented as controls. Both members of the pairs in the supplemented group had access to the yolk and consumed it; however, due to the large amount we provided, both members of each pair were able to take in a substantial quantity (pers. obs.). While providing supplementation with yolks from quail would have been ideal, the supplementation protocol required a large amount of total yolk, and we did not have access to enough quail yolks to supplement all pairs daily. We chose chicken yolks instead because our goal was to provide the birds with lipid and protein supplementation that may elevate body condition and increase circulating concentrations of both protein and lipid that may ultimately be used to make yolk precursors. In addition, a comparison of chicken and quail egg yolk showed that they have very similar compositions (Tolik et al., 2014).

**Table 1:** Nutrient composition for quail diet

Ingredient	Formulated % of diet by weight
Corn	57.00
Soybean meal	20.40
Limestone	4.70
Salt	0.60
Dicalcium phosphate	1.30
Trace mineral	0.08
Vitamins	0.25
dl‐Methionine	0.05
Fish meal	5.00
Alfalfa meal	5.00
Wheat midds	5.00
Soybean oil	0.68
Safflower seed oil	0
Flaxseed oil	0
l‐Lysine	0
Threonine	0
Coban 90	0
Solka flock	0

**Figure 1:**
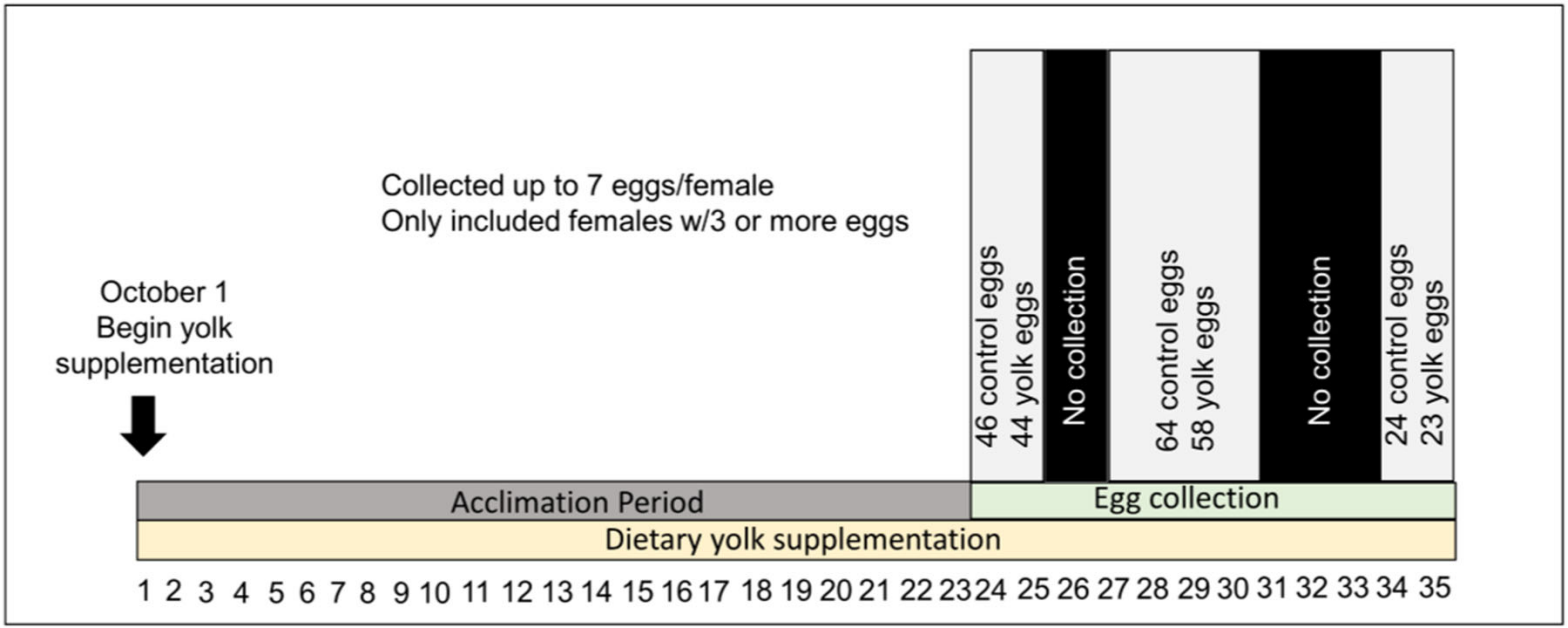
A diagram of the experimental timing employed. Birds were given dietary supplementation with hard‐boiled chicken yolks.

After 3 weeks of dietary supplementation, we began egg collections. We waited this 3‐week period because quail eggs can take anywhere from 4 to 7 days to complete RYD (Bacon & Koontz, 1971); we wanted to be sure that all birds had acclimated to the treatment for at least 2 weeks and all eggs we measured were influenced by the dietary supplementation. We collected a total of at least three (range: 3−7) eggs from each female. Because this study was part of another one, we were not able to monitor the full laying sequence and collected eggs within three 2−3‐day time frames over a 12‐day period (see Figure 1). Eggs were immediately frozen after collection to await yolk ring analyses. Dietary supplementation with chicken egg yolk continued throughout the duration of egg collection. Finally, at the completion of the experiment, after 6 weeks of supplementation, we again measured body weights and tarsus lengths for all females. During the course of the experiment, four females were removed from the experiment due to illness or death. We also pre‐established a minimum egg production for inclusion in the experiment of three eggs per week. While most birds lay eggs daily, there was variation in our population in the laying rate, and five control and five yolk pairs did not lay eggs regularly enough to include them in the analyses. This left 25 breeding pairs in the control group and 21 in the dietary yolk group.

### 2.2 Female body condition index

We calculated the female body condition index (BCI) using the residuals of mass:tarsus length collected before and after the yolk supplementation (Labocha & Hayes, 2012). Because BCI can change over a month of active egg‐laying, we used the average of the initial and final BCI in our analyses (hereafter called BCI). We also calculated the change in BCI from the first to the second measurement.

### 2.3 Measurement of ovarian follicle growth

From each pair, we obtained eggs collected over all 7 days (see Figure 1 for experimental timing), incubated them for 2 days to obtain genetic information for another experiment, and then immediately froze the eggs. This short period of incubation results in only a small blastocyst that is not large enough to disrupt the yolk rings based on pilot experiments. We then weighed the eggs using a digital scale (VWR‐500p, accuracy = 0.01 g). To separate yolks from the eggs for staining, we first covered eggs with dry ice for approximately 5 min to ensure solid freezing and then hit each egg with the back of a knife to crack off the shell and albumen. The albumen cracked off completely and the result was usually an isolated, intact egg yolk with no albumen attached. In some cases, the yolk shattered when we were cracking the eggs, so the final intact samples included a total of 198 egg yolks, with 3−7 yolks from every female. We then placed the yolk in a 50 ml conical tube containing 4% formalin and incubated it in a water bath set at 60°C for 6 h, after which we removed the formalin, covered the yolk with 3% potassium dichromate, and incubated again at 60°C for 24 h. These methods were loosely based on those described in Grau (1976), but after many test yolks came out too dark to be read, we had to reduce the concentration of potassium dichromate from 6% to 3%, alter the timing from the 12 h:12 h incubation periods described in Grau (1976), and keep the yolks whole during the staining step rather than cutting them in half as was described previously.

**Figure 2:**
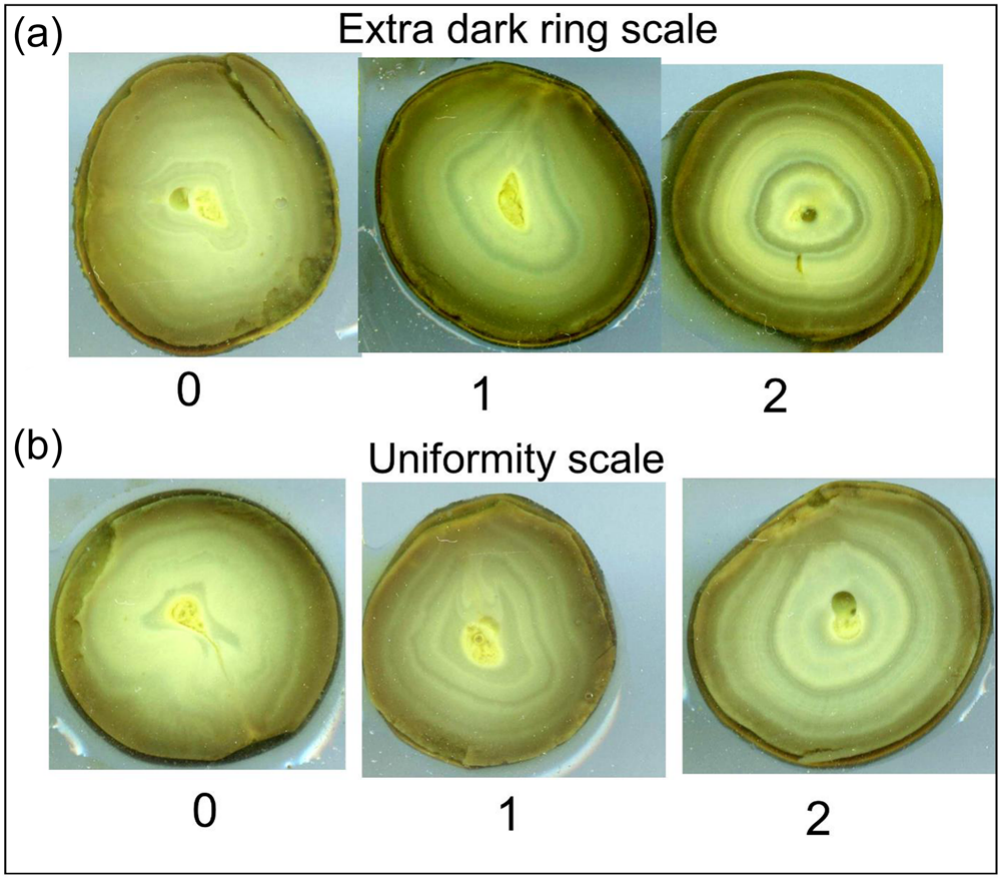
Procedures for ranking and measuring stained quail yolks, including (a) rankings for the presence of extra dark rings. Yolks were assigned a score of 0 if no extra dark rings were present, 1 if a single extra dark ring was present, and 2 if two or more extra dark rings were present; (b) rankings of the uniformity of yolk rings. Yolks with rings that were almost perfectly uniform were assigned a rank of 2, those with one irregular ring were assigned a rank of 1, and those with many irregular rings were assigned a rank of 0.

After staining, we then cut the yolks in half, dipped them in water to rinse them and also because wet yolks produced much better images than dry yolks. We then immediately scanned them using a high‐resolution scanner (Epson Perfection V550 Photo; Epson America). We used NIH's ImageJ software (http://imagej.nih.gov/ij/) to increase the contrast to approximately 0.3% saturation to increase the visibility of rings and counted all dark and light rings that were visible on each yolk. We also used the ImageJ measurement tool to manually measure each light and each dark ring in each yolk and obtained a mean thickness for all light and dark rings. While counting yolk rings, we noticed that some yolks stood out because they had 1−5 rings that stained extra dark compared to all other rings in the yolk, so we scored all yolks on a scale of 0−2, with 0 representing yolks with no extra dark rings, 1 representing yolks with 1 extra dark ring, and 2 representing yolks with 2 or more extra dark rings (Figure 2a). We also noticed that some of the yolks contained rings that were quite uniform, while others had rings that were not circular, but appeared squished. As a result, we ranked yolks on a uniformity scale; yolks scoring 2 had rings that were almost perfectly uniform, yolks scoring 1 had one ring that was not circular, and yolks scoring 0 appeared skewed through much of the yolk (Figure 2b).

### 2.4 Statistical analyses

First, we calculated the averages of each yolk measure for each female (over 3−7 eggs per female). We chose to conduct the analyses this way because, when running the analyses using individual data points, we ran into problems with singular fit issues for some of the variables, preventing us from running the analyses as individual data points. These issues were not present when we ran the data using averages. Additionally, the variables for which we *could* run analyses using individual data points gave similar significant results either way. So we felt confident that using averages was the best and most conservative way to test the impacts of treatment on the measured yolk variables. We then tested each variable for normality using the Shapiro−Wilk tests. Average BCI and change in female BCI were non‐normally distributed and were log‐transformed to achieve normality. The average scores for the presence of extra dark rings were also non‐normally distributed, so this variable was analyzed using a generalized linear mixed model (see below). All of the remaining variables (yolk diameter, yolk mass, average number of rings, uniformity, and the thickness of light and dark rings) were normally distributed and did not require any transformations.

We first tested whether treatment influenced the change in female BCI and whether the average of the initial and final female BCI was significantly related to yolk diameter using general linear models. Because treatment did not influence the change in BCI, we did not include it in further analyses. Further, since BCI was not related to treatment (see below for these results), we were able to incorporate the effects of both treatment and average female BCI into the same model for further analyses. We tested whether treatment and/or BCI influenced the following using general linear models:

– rates of yolk deposition (i.e., the number of yolk rings);
– the presence of extra dark rings;
– the presence of thick dark rings;
– yolk uniformity;
– yolk diameter;
– yolk mass;
– egg mass;
– mean thicknesses of light and dark rings.

For these models, we used treatment, the average BCI, and the interaction between treatment and average BCI as fixed factors. The average scores for the presence of extra dark rings were not normally distributed. Because this analysis was run with the averages of the “yes/no” scores, the distribution of these averages was also not binomial, but instead fit an exponential distribution based on a distribution test in JMP (AICc = 39.81, weight = 99.56). So we ran the analyses using a generalized linear model using an exponential distribution and a reciprocal link, which can be used for analyses with generalized linear mixed models (Tuerlinckx et al., 2006). Finally, we tested whether the number of yolk rings (follicle growth rates) was related to the diameter of the follicles and the thicknesses of light and dark rings using a general linear model, including mean thickness of light rings and dark rings and yolk diameter as fixed factors. We used JMP© V. 14 statistical software (SAS Institute, Inc.) for all statistical analyses.

## 3 RESULTS

### 3.1 Effects of dietary supplementation on body condition

There was no difference in initial (*F*_1,70_ = 0.12, *p* = 0.73), final (*F*_1,51_ = 0.59, *p* = 0.45), or average BCI (*F*_1,51_ = 0.64, *p* = 0.43) between the dietary treatment groups. Dietary supplementation with hard‐boiled chicken egg yolk did not influence the change in female body weight (*F*_1,51_ = 0.09, *p* = 0.77).

### 3.2 Relationships between female BCI and follicle growth

**Figure 3:**
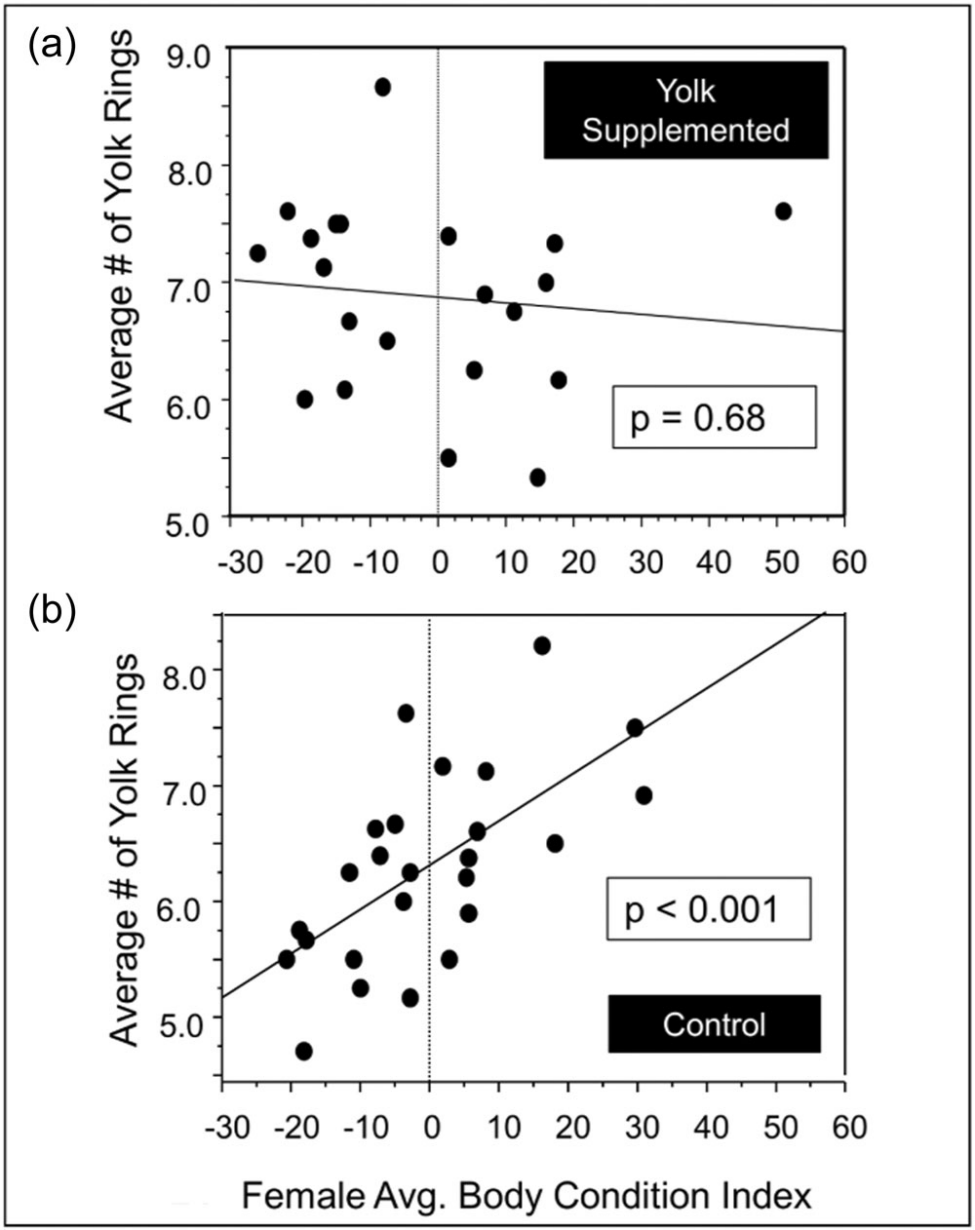
Influences of female condition on follicle growth rates. Shown is the relationship between the initial female body condition and the average number of rings in yolks produced by control (*n* = 25) and yolk‐supplemented females (*n* = 21). Body condition was calculated as the residuals of the regression of mass:tarsus length. Regression analyses indicated that in the control group, body condition before the experiment began was positively related to the number of yolk rings (i.e., females with high body condition produced slower‐growing follicles) (*R* ^2^ = 0.26, *p* = 0.01) but there was no such relationship in the yolk‐supplemented group (*R* ^2^ = 0.02, *p* = 0.53).

We next examined whether the female's BCI related significantly to the growth rates of the ovarian follicles (i.e., a larger number of yolk rings = a slower rate of growth). There was an overall significant positive relationship between BCI and the number of rings in yolks (*F*_1,44_ = 4.79, *p* = 0.02, Table 2), but a significant interaction effect of treatment and BCI indicated that this relationship differed between the two treatment groups (*F*_1,44_ = 9.50, *p* = 0.004, Table 2). When the relationship between BCI and the number of yolk rings was examined in the two treatment groups separately, there was a significant positive correlation between BCI and the number of follicle growth rings in control females (*F*_1,23_ = 15.39, *p* = 0.0007, Figure 3a), but this relationship disappeared when the diet was supplemented with hard‐boiled egg yolks (*F*_1_,_19_ = 0.23, *p* = 0.63, Figure 3b). This indicates that, normally, females with higher BCIs grow follicles more slowly, and that supplementing the diet with egg yolks disrupts this relationship, likely by decreasing growth rates in the females with low BCIs.

**Table 2:** Model output for factors influencing yolk characteristics

Independent variable	Number of rings			Extra dark rings			Uniformity			Yolk diameter		
	ES	*F*	*p*	ES	*Χ* ^2^	*p*	ES	*F*	*p*	ES	*F*	*p*
Treatment	0.12	7.74	0.008	0.23	11.06	0.0009	0.02	1.35	0.25	0.009	0.39	0.54
F. BCI	0.05	5.79	0.02	0.03	3.63	0.057	3.82 × 10^−6^	0.30	0.58	0.061	2.51	0.12
Treatment × F. BCI	0.15	9.50	0.004	0.004	2.69	0.10	0.14	6.81	0.01	0.001	0.04	0.83

Abbreviations: ES, effect size; F. BCI, female body condition index.

### 3.3 Effects of dietary supplementation on follicle growth

**Figure 4:**
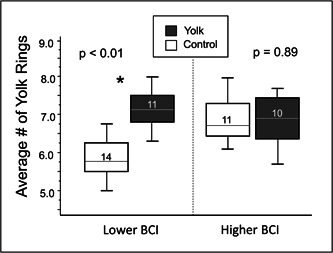
Influences of yolk‐supplementation on the average number of yolk rings deposited by females in low and high initial body conditions. Females with body condition indices (BCI) below the median value were assigned to the low condition group (*n* = 25), while females with BCIs above the median value were assigned to the high condition group (*n* = 21). A general linear model indicated that for females with low body conditions, treatment with yolk significantly increased the number of yolk rings (i.e., decreased follicle growth rates) compared to controls (*F*_1,24_ = 15.52, *p* < 0.001), while there was no difference between supplemented and control females that began the experiment with a high body condition index (*F*_1,20_ = 1.77, *p* = 0.20). Error bars represent standard errors. BMI, body mass index.

We next examined whether dietary supplementation with egg yolk could change the rates of follicle growth. There was an influence of dietary yolk supplementation on the number of growth rings (*F*_1,44_ = 7.74, *p* = 0.008, Table 2), but the significant interaction effect between treatment and BCI (*F*_1,44_ = 9.50, *p* = 0.004, Table 2) indicates that the effects of treatment depended on the BCI of the female. To further examine this interaction effect, we split females into higher (above the median log‐transformed value, *n* = 22) and lower (below the median log‐transformed value, *n* = 24) groups, and tested the influence of dietary supplementation on the number of yolk rings in females with “low” and “high” body condition using a general linear model. Treatment significantly increased the number of yolk rings in birds with lower BCIs (*F*_1,23_ = 12.78, *p* = 0.002, Figure 4), meaning that providing dietary yolk supplements triggered these females to slow their rates of follicular growth. In contrast, there was no influence of treatment in the yolks of females with higher BCIs (*F*_1,21_ = 0.02, *p* = 0.89, Figure 4).

### 3.4 Effect of dietary supplementation and BCI on other egg and yolk characteristics

**Figure 5:**
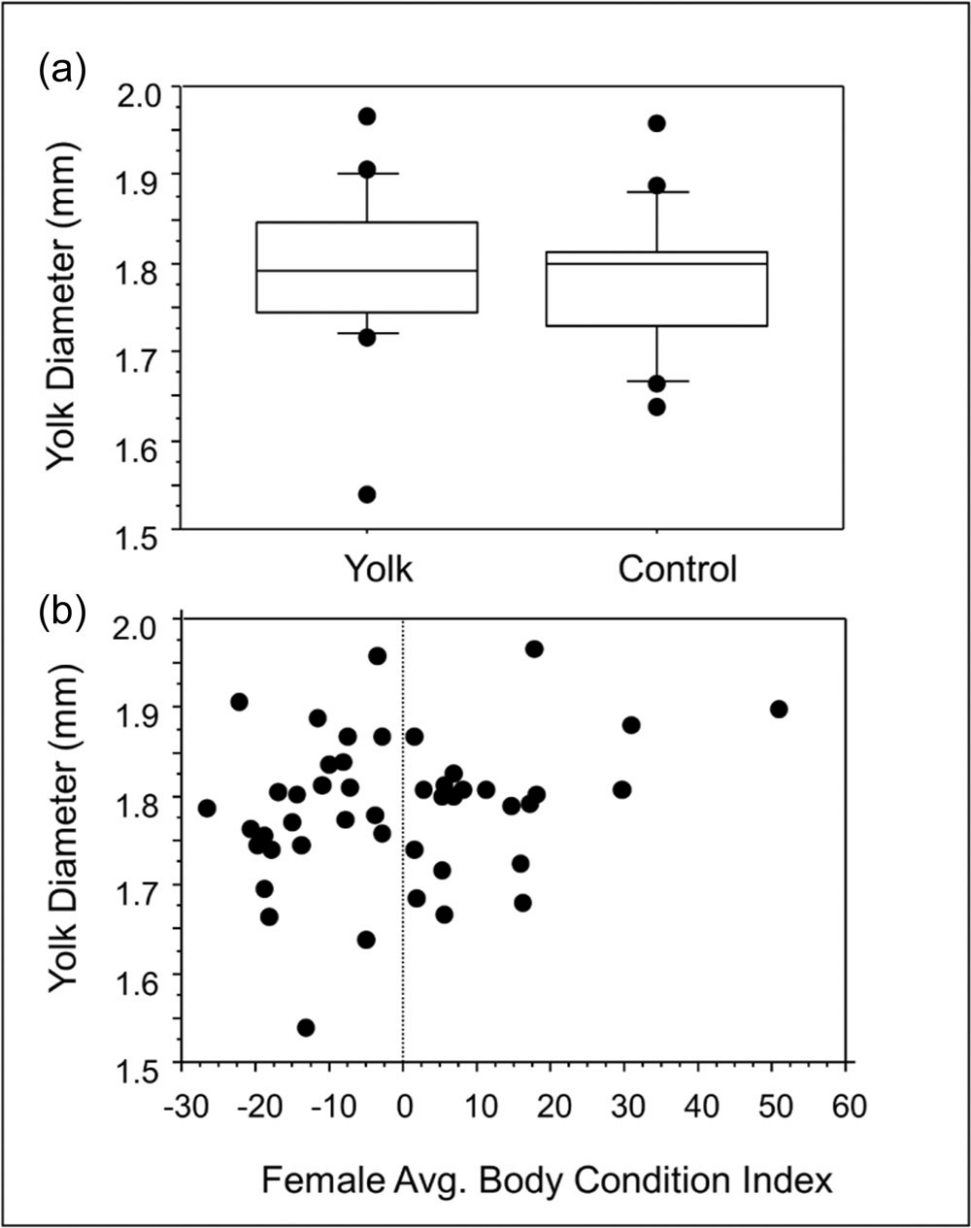
Impacts of dietary treatment on yolk diameters (a) and relationship between female average BCI and yolk diameter (b).

We next examined the effects of dietary yolk supplementation on the presence of extra dark rings in the yolk and on yolk uniformity (Table 2). Birds that were given dietary yolk supplementation had dark rings that were extra dark in their yolks more often (*χ* ^2^_1,44_ = 11.06, *p* = 0.0009) (Figure 5) and there was a significant interaction between treatment and BCI on uniformity of the yolk (*F*_1,44_ = 6.81, *p* = 0.01). When this relationship was explored further by splitting birds into lower and higher BCI groups as above, supplying supplemental yolk disrupted uniformity only in the birds with higher BCIs (*F*_1,19_ = 6.31, *p* = 0.02), while there was no influence of treatment on yolk uniformity in birds with lower BCIs (*F*_1,23_ = 0.04, *p* = 0.83). There was no impact of treatment on yolk diamater (Table 2 and Figure 5a) nor was there a relationship between yolk diameter and female BCI (Table 2 and Figure 5b).

**Figure 6:**
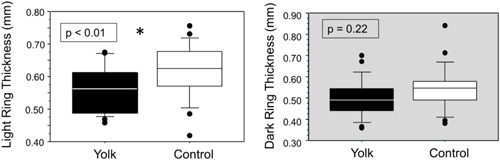
Influences of dietary treatment on the thicknesses of light rings (a, deposited during the night) and dark rings (b, deposited during the day). Yolk supplementation significantly decreased the thickness of light ring, but did not influence the thickness of dark rings.

Given that we saw differences in the numbers of yolk rings between treatments and in relation to BCI but not yolk diameters, the next step was to examine the impacts on yolk ring thicknesses. We looked at how dietary treatment influenced the thicknesses of the rings deposited during the day (dark rings) and the night (light rings). Dietary treatment and female significantly influenced the thickness of the light rings (deposited at night) in the yolks (Treatment: *F*_1,44_ = 7.58, *p* = 0.0009, Table 3). Females supplemented with egg yolk had thinner light rings, indicating slower growth at night. Average BCI did not significantly relate to the thickness of the light rings (*F*_1,44_ = BCI: *F*_1,44_ = 2.60, *p* = 0.11) nor was there a significant interaction between treatment and average BCI (*F*_1,44_ = 1.54, *p* = 0.22). Neither dietary treatment nor BCI was significantly related to the thickness of dark rings (deposited during the day) (Treatment: *F*_1,44_ = 1.37, *p* = 0.24; BCI: *F*_1,44_ = 0.02, *p* = 0.89, Figure 6b), nor were they related to yolk diameter (Treatment: *F*_1,44_ = 0.39, *p* = 0.54; BCI: *F*_1,44_ = 2.51, *p* = 0.12). Treatment did not influence egg or yolk mass (egg mass: *F*_1,40_ = 0.06, *p* = 0.81; yolk mass: *F*_1,39_ = 1.32, *p* = 0.26), but average BCI was significantly related to both (egg mass: *F*_1,40_ = 10.32, *p* = 0.003; yolk mass: *F*_1,39_ = 15.04, *p* = 0.0004). There was no interaction effect on either variable (egg mass: *F*_1,40_ = 1.90, *p* = 0.18; yolk mass: *F*_1,39_ = 0.02, *p* = 0.89, Table 3).

**Table 3:** Model outputs for factors influencing yolk ring thickness, yolk mass, and egg mass

	Mean light ring thickness			Mean dark ring thickness			Yolk mass			Egg mass		
Independent variable	ES	*F*	*p*	ES	*F*	*p*	ES	*F*	*p*	ES	*F*	*p*
Treatment	0.15	7.58	0.0009	0.03	1.37	0.24	0.02	1.11	0.30	0.002	0.10	0.75
F. BCI	0.04	2.60	0.11	0.007	0.02	0.89	0.29	15.26	0.0004	0.24	11.35	0.002
Treatment × F. BCI	0.03	1.54	0.22	0.08	3.81	0.057	0.001	0.06	0.81	0.04	2.30	0.04

Abbreviations: ES, effect size; F. BCI, female body condition index.

### 3.5 Characteristics of yolks

The number of yolk rings was not related to the yolk diameter (*F*_1,44_ = 2.29, *p* = 0.14), but was related to the thickness of dark and light rings (dark: *F*_1,44_ = 9.52, *p* = 0.004, light: *F*_1,44_ = 18.50, *p* < 0.001). Both light and dark rings were significantly thinner when there were more rings in the yolk. While ovulation is not necessarily dependent on the size of the yolk (Etches et al., 1983), it appears that these follicles are taking longer to reach the point when they can respond adequately to the luteinizing hormone.

## 4 DISCUSSION

We initially hypothesized that supplementing quail diets with hard‐boiled egg yolk would provide birds with the yolk precursors they need to increase rates of yolk deposition and, as a result, follicle growth. We also predicted that females with larger fat reserves, as indicated by a higher BCI, would grow follicles at a faster rate because they would have more yolk precursors available to them to deposit into growing follicles. Instead, we saw the opposite. Females with higher BCIs had more rings in their yolks while the diameter of their yolks did not differ from those of birds with lower BCIs. This indicates that birds with high BCIs grew their ovarian follicles more slowly. Further, supplementing females that had low BCIs with dietary egg yolk decreased the rates at which they grew their follicles to rates that were comparable to the females with high BCIs (Figure 3).

Since we saw variation in the number of follicle growth rings but not in yolk diameters between females with high and low BCIs and between treatment groups, we would expect to see differences in the thicknesses of the rings within the yolks. Our analyses indicate that variation in follicle growth rates arose from differences in rates of yolk deposition that occurred during the dark phase. During RYD, females deposit more pigmented yellow darker staining yolk during the day and less‐pigmented (often called “white”) lighter staining yolk during the night. In eggs laid by control females, the light rings that were deposited at night (light rings) were, on average, thicker than the dark rings that were deposited during the day (light: 0.62 cm, dark: 0.50 cm). This means that under normal conditions, more yolk is deposited during the night compared to during the day, a result that has also been shown in previous studies of follicle growth in laying hens (Moran, 1987; Zakaria et al., 1984). In the current study, supplementing the diet with egg yolk decreased the disparity between the thicknesses of light and dark rings (light: 0.55 cm, dark: 0.54 cm) by decreasing the rate of deposition at night.

The question, then, is why would female quails with a higher BCI, and females with high access to yolk precursors, deposit thinner rings each night compared to quails in lower body condition and/or with lower availability of yolk precursors? Successful deposition of yolk requires the liver to shift from producing generic VLDL to producing VLDLy, which is rich in triacylglycerol, is more resistant to peripheral metabolism, and is half the size of generic VLDL. These characteristics allow the VLDLy to make it to the oocyte and to be taken up by the VLDL receptors on the oocyte (Walzem et al., 1999). Also required is the successful deposition of those particles by receptor‐mediated endocytosis (Bujo et al., 1997; Nimpf et al., 1988). The components of the yellow yolk are formed from intestinal contents and are deposited during the day to form the darker rings. White yolk (which forms the lighter rings deposited at night) is deposited after the intestines are empty, so its components are thought to originate from vitellogenin reserves in the bird (Moran, 1987) and through the synthesis of fat from carbohydrates and proteins in the diet (Warren & Conrad, 1939). The decreases we saw in the rate of yolk deposition would have to occur at one of these steps.

It is possible that the production of VLDLy and/or its uptake by the oocyte were influenced by the feeding pattern of the dietary yolk supplementation. In this experiment, the supplemental yolk was provided once per day in the morning, and birds consumed the boiled egg yolk by noon. Perhaps the rapid intake of this high‐calorie supplementation prevented the birds from feeding during the remainder of the day, which might be reflected in the production of VLDLy at night. This does not seem likely, however, because the slow follicle growth rates were observed not only in females supplemented with yolk but also in control females with higher BCIs. These females would not have had yolk supplements as triggers to take in large quantities of food in the morning. Instead, food was always available to the females and we observed feeding by females in all treatment groups in the afternoon on many occasions, so we do not believe females limited their feeding behavior to the morning hours. Additionally, work in laying hens suggests that the rate of vitellogenesis is not so acutely responsive to rapid changes in food intake, as it took 3 days of food deprivation to reduce the rate of vitellogenesis in those birds (Sekimoto et al., 1990). In fact, previous studies in both laying hens and European starlings have shown no diurnal variation in the amount of vitellogenin in the bloodstream and that vitellogenin concentrations in the blood were extremely high and unlikely to be depleted at night (Challenger et al., 2001; Redshaw & Follett, 1976). This indicates that differences in the rate of yolk deposition between day and night were not likely the result of variation in the amount of VLDLy available.

Instead, the effects we saw might result from diurnal variation in the rate of uptake by the oocyte instead. Schneider (2008) suggested that transient ligand binding capacity within the oocyte may undergo a diurnal rhythm that can account for the difference in yolk deposition rates. Thus, it is possible that the addition of egg yolk to the diet or a high BCI in our study decreased the ability of oocytes to take up yolk, particularly during the nighttime hours. Walzem et al. (1994) found that overfeeding of laying hens increased the VLDL diameter, and prevented the uptake of the lipoproteins by the oocyte. Because the spacing of the granulosa basal lamina acts as a filter, allowing in only molecules below a particular size (Griffin et al., 1984), this could decrease the amount of yolk precursors that are allowed to pass into the yolk, decreasing follicle growth rates as a result. If a similar effect occurred in our quail, this could explain the decrease in the rate of yolk deposition in birds that had either high‐fat reserves or were supplemented with dietary egg yolk. However, since yolk was supplemented in the morning, why did we see the effect in nighttime yolk deposition? A previous study showed that a fat‐soluble dye fed to laying hens took 7−11 h to be deposited into the yolk (Lacassagne, 1960). If the timing is similar in quail, then the dietary yolk that they were fed in the morning could have been in circulation and ready for deposition around nighttime. Similarly, females with high body fat reserves may have more VLDL that are larger in diameter in circulation at night than those that are leaner and rely mostly on dietary sources. This remains to be tested.

It is also possible that high body condition index and supplementing with hard‐boiled egg‐yolk triggers reproductive dysfunction within the female quails, limiting their ability to deposit yolk into follicles during RYD. In the current study, we fed the yolk supplements to quail for 3 weeks, which is likely a longer duration than a wild bird would have access to supplemental fat or protein beyond the normal diet. In previous studies where resource availability was positively correlated with yolk size, resource availability within 3 days was the most important predictor (Ardia et al., 2006). In addition, studies of wild birds kept in captivity have shown that captive diets and conditions often lead to obesity and fatty liver disease (Wadsworth et al., 1984); both can negatively impact reproductive function. As a result, while we did not see an effect of our dietary supplements on body condition, it is possible that, like other birds in captivity, our captive females were already at the upper limits of their body weight, which would have prevented such an effect.

Our study is not the first case where the addition of fat to an avian diet resulted in the deposition of a particularly thin ring of yolk. Grau et al. (1977) fed Japanese quails a single dose of petroleum oil and examined the resulting effects on yolk rings. Yolks in eggs laid by oil‐fed quail contained layers that stained extra dark, much like the extra dark layers that were more prevalent in eggs from our yolk‐supplemented birds. In addition, less than the normal amount of yolk was deposited the first night after the petroleum oil was fed by Grau and colleagues. The authors suggested that a toxic component of the petroleum oil might have disrupted normal liver function, though they did not elaborate on how this might have occurred. In addition, studies in broiler hens, in which reproductive dysfunction is prevalent, showed that feed restriction resulted in faster rates of yolk deposition (Watson, 1975; Yu et al., 1992 but see Nestor et al., 1970; Robinson et al., 1993).

Studies in chickens have shown that, while egg production requires energy, a positive energy balance can quickly result in impaired reproductive function (reviewed in Walzem & Chen, 2014). Of particular relevance to the current study, overfeeding of hens induces lipotoxicity (Chen et al., 2006). Obesity and high‐calorie diets are also linked to dysregulation of estrogen production (Gardner & Young, 1972; Whitehead et al., 1993). Estrogens are important in the initiation of vitellogenesis in the liver and while the dosage of estrogen does not appear to influence the rate of vitellogenesis (Gruber et al., 1976), the uptake of yolk proteins is influenced by hormonal signals (e.g., FSH signaling, Palmer & Bahr, 1992) that can be downregulated by high concentrations of estrogen in circulation (Farner & Wingfield, 1980). In fact, the treatment of European starlings with estradiol reduced yolk protein and lipid content. Finally, our study yielded another piece of evidence that supports this possibility. Some of the yolks we stained contained rings that were not uniform; a phenomenon that has been previously documented in Adélie penguins and has been proposed as evidence that atresia had begun in those follicles before ovulation (Astheimer & Grau, 1985). In our study, yolk supplementation increased the incidence of nonuniform egg yolks only in eggs produced by females with high body condition. Perhaps high levels of body fat combined with yolk supplementation drives growing follicles into a state of atresia, which would provide further evidence that too much fat in circulation can dysregulate processes involved in oocyte growth and survival.

Given these effects, it would be interesting to test whether supplementing with smaller amounts of yolk influences results in the same changes that we saw in the current study. We did not measure how much yolk was consumed by each female each day. Dishes containing 18 g of yolk were put into cages in mid‐morning, and were retrieved approximately 5 h later. Based on observations, it took at least 2 h for the pairs to consume all of the yolk, and by 5 h after the dishes were placed, all of the yolk was consumed each day. This indicates that there was enough time for both members of the pair to consume yolk and that it was not too much yolk to consume; however, since both the male and the female were both in the cage, it was unclear whether we hit the upper limit of what females could consume. Perhaps supplementation with a smaller amount would have a different influence on rates of yolk deposition. Future studies should address this possibility.

In wildlife studies, we tend to think that more food is better, and that higher body condition indices lead to higher success in reproduction and other life‐history variables. The results of our study suggest that we need to further examine the relationship between body condition and egg quality. Given that important physiological modulators are deposited along with yolk precursors (Grindstaff et al., 2003; Groothuis & von Engelhardt, 2005; Navara et al., 2006), it is now important to determine whether body condition and food availability influence the relationships between follicle growth and deposition of these biologically active materials. It is also important to quantify the lipid and protein content of slow and fast‐growing yolks, determine the influences of food deficiency on follicle growth, and test whether the growth rate of follicles influences the growth rates of embryos that will grow within the eggs. This study indicates that we need to consider optimal body conditions and resource intakes when assessing reproductive needs in birds and other animals.

## AUTHOR CONTRIBUTIONS

Kristen J. Navara designed the study and performed the statistical analyses, Kylie Graden collected data and performed the experiment, and Mary T. Mendonça participated in the experimental design.

## CONFLICT OF INTEREST

The authors declare no conflict of interest.

## ETHICS STATEMENT

This study was approved by the UGA Institutional Animal Care and Use Committee (A2017 10‐019‐Y1‐A1) and was performed in compliance with the US National Research Council's Guide for the Care and Use of Laboratory Animals, the US Public Health Service's Policy on Humane Care and Use of Laboratory Animals, and Guide for the Care and Use of Laboratory Animals.

## Data Availability

Data will be made publicly available upon publication in the Dryad Data Repository.
